# Reduction of blood loss in primary hip arthroplasty with tranexamic acid or fibrin spray: A randomized controlled trial

**DOI:** 10.3109/17453674.2011.623568

**Published:** 2011-11-25

**Authors:** Jamie S McConnell, Sandeep Shewale, Niall A Munro, Kalpesh Shah, Angela H Deakin, Andrew WG Kinninmonth

**Affiliations:** ^1^Royal National Orthopaedic Hospital, Stanmore, Middlesex; ^2^Monklands Hospital, Airdrie; ^3^Golden Jubilee National Hospital, Clydebank, UK

## Abstract

**Background and purpose:**

Previous studies have shown that either fibrin spray or tranexamic acid can reduce blood loss at total hip replacement, but the 2 treatments have not been directly compared. We therefore conducted a randomized, controlled trial.

**Patients and methods:**

In this randomized controlled trial we compared the effect of tranexamic acid and fibrin spray on blood loss in cemented total hip arthroplasty. 66 patients were randomized to 1 of 3 parallel groups receiving (1) a 10 mg/kg bolus of tranexamic acid prior to surgery, (2) 10 mL of fibrin spray during surgery, or (3) neither. All participants except the surgeon were blinded as to treatment group until data analysis was complete. Blood loss was calculated from preoperative and postoperative hematocrit.

**Results:**

Neither active treatment was found to be superior to the other in terms of overall blood loss. Losses were lower than those in the control group, when using either tranexamic acid (22% lower, p = 0.02) or fibrin spray (32% lower, p = 0.02).

**Interpretation:**

We found that the use of tranexamic acid at induction, or topical fibrin spray intraoperatively, reduced blood loss compared to the control group. Blood loss was similar in the fibrin spray group and in the tranexamic acid group.

ClinicalTrials.gov identifier: NCT00378872

EudraCT identifier: 2006-001299-19

Regional Ethics Committee approval: 06/S0703/55, granted June 6, 2006

Several different blood conservation methods have been adopted in lower limb arthroplasty. This study investigates the hemostatic effects of tranexamic acid and fibrin spray. The effects of these medications have been reported in isolation, but there have been few reports comparing their efficacy in the context of total hip replacement.

Tranexamic acid is a synthetic amino acid that competitively inhibits plasminogen, leading to reduced fibrinolysis locally (Ho and Ismail 2003). Fibrin spray is composed of active ingredients derived from human blood products, mainly fibrinogen and thrombin. These components are activated when mixed, forming fibrin which crosslinks directly with tissue collagen. Both of these therapies have been shown to be safe and effective in reducing blood loss in hip arthroplasty (Ho and Ismail 2003, Wang et al. 2003, Johansson et al. 2005). Differences in methodology between previous studies mean that it is difficult to draw comparisons between the two medications; such comparisons have not been widely reported in the context of cemented hip arthroplasty.

We compared the effects of fibrin spray and tranexamic acid in cemented hip arthroplasty with total blood loss as the endpoint. We hypothesized that both treatments would have equal effectiveness.

## Patients and methods

This was a prospective, single-center, multi-surgeon, single-blind controlled trial with equal randomization. It was conducted in the United Kingdom with approval from the West Glasgow Research Ethics Committee and the Medicines and Healthcare products Regulatory Agency, and it was performed in compliance with the Helsinki Declaration of 1975, as revised in 2000.

Patients were recruited June 2006 through May 2008. Each patient underwent a single joint replacement. Eligible patients were approached and given information sheets at preoperative assessment clinics. Patients were eligible if they were scheduled to undergo elective primary unilateral cemented hip arthroplasty. They were excluded if they were taking anticoagulant medication or had a known coagulopathy, as such patients would have been predisposed to higher blood loss. Patients were also excluded if there were contraindications to giving the medications in the study: known allergy to the medications used, including allergy to aspirin; previous reaction to blood products; ethical/religious objection to receiving blood products; or previous thromboembolism. Informed consent was obtained on admission to hospital. 4 patients who were assessed for eligibility were excluded because they were awaiting uncemented hip arthroplasty. 1 was excluded because there was no fibrin spray in stock on the day of the operation. The study protocol required that if patients were excluded because of protocol violations, they would be replaced by newly-recruited patients.

Patients were randomized to 1 of 3 treatment groups: (1) tranexamic acid (Cyklokapron; Meda Pharmaceuticals, UK), (2) fibrin spray (Quixil; Omrix Biopharmaceuticals, Belgium), or (3) neither (control). This was to assess the efficacy of each treatment against the other and against a control group, with total calculated blood loss as the endpoint. Patients were randomized to treatment groups by use of opaque, sealed envelopes. These were opened by the anesthetist immediately before anesthetic induction and they were not handled by the surgeon. This was a single-blind study: it was not possible to blind the surgeon to the application of the fibrin spray, as it creates a visible film on the operative wound when applied. The patients and all other staff involved in patient care remained blinded as to the treatment used. Blinding was maintained until statistical analysis of data, which was undertaken by an independent statistician.

Criteria for stopping the study were the completion of data collection, or if any of the treatment groups were found to have a substantial increase in adverse clinical outcome(s) or rate of postoperative complications.

Blood tests for full blood count, urea and electrolytes, clotting, and cross-match were obtained on admission. The patient's weight, height, sex, and preoperative hematocrit were recorded. Each of the treatment groups had similar mean BMI and preoperative hematocrit (Table).

**Table:** Patient data

	M	F	Hct (pre)	BMI
Control	9	13	0.40	28.49
Fibrin spray	5	17	0.40	28.02
Tranexamic	7	15	0.40	27.04
All	21	45	0.40	27.85

A standardized anesthetic was used in all cases, consisting of combined regional anesthesia by epidural and general anaesthesia by target-controlled infusion of propofol. All hip replacements and data collection were performed at a single site (Golden Jubilee National Hospital, Clydebank, Scotland). A cemented Exeter hip prosthesis was used in all cases (Stryker Howmedica Osteonics, NJ). No drains were used.

Patients randomized to the fibrin group received 10 mL of topical fibrin spray, given in divided doses throughout the operation. Patients in the tranexamic acid group were given a single 10 mg/kg dose of tranexamic acid as an intravenous bolus at the start of the surgical procedure. All patients received the intended treatment to which they were allocated.

A standard postoperative rehabilitation regime was used. All patients received standardized DVT prophylaxis as recommended by local guidelines at the time of the study. This included graduated compression stockings, early mobilization, and 150 mg of aspirin by mouth for 35 days postoperatively. Hematocrit measurement was repeated on the second postoperative day. No patients were lost to follow-up and data collection was completed for all participants.

The patient's total blood volume (PBV) was calculated, taking account of sex, height, and weight. Multiplication of PBV by hematocrit (Hct) will give total red cell volume in mL (Nadler 1962). Any change in red cell volume can therefore be calculated from the change in Hct. Any volume transfused or re-transfused is added to yield the true blood loss (Sehat et al. 2004). This can be summarized by the formula:

True blood loss = ((PBV × change in Hct) / mean Hct ) + volume transfused

We used this method of calculation because it takes account of “hidden” losses (Sehat et al. 2004), e.g. postoperative hematoma formation (Benoni et al. 2001), which can be greatly underestimate when blood loss is recorded from suction drains or swabs (Sehat et al. 2004).

### Statistics

For calculation of power, we considered blood loss of 250 mL to be a clinically significant amount. To detect this difference at 80% power and at the 5% significance level (α = 0.05) assuming an expected standard deviation (SD) of 290 mL for each group (Claeys et al. 2007), we required a sample size of 22 patients per group. Recruitment continued until a total of 66 patients were enrolled. Data for the 3 treatments (tranexamic acid, fibrin spray, control) were analyzed using the Kruskal-Wallis test; pairwise comparisons were then performed using the Mann-Whitney U test. Analysis was performed with originally assigned groups of 22 patients per treatment group. Data were analyzed with Minitab software version 15 (Minitab Inc., State College, PA).

## Results

No adverse outcomes were reported during the trial, so the trial ended when data collection was complete. Overall median intraoperative blood loss as measured from swabs and suction was 400 mL with an interquartile range (IQR) of 238 mL. One patient (in the fibrin spray group) was transfused with 2 units of packed red cells, and this was corrected for when calculating total blood loss. Calculated median total blood loss in the control group was 1.20 L (95% CI: 0.82–1.4; IQR: 0.50). Loss in the tranexamic acid group was median 0.93 L (CI: 0.69–1.1; IQR: 0.35). In the fibrin spray group, median loss was 0.82 L (CI: 0.72–1.1; IQR: 0.33).

There was a statistically significant difference in blood loss between the 3 groups (Kruskal-Wallis test, p = 0.02). On pairwise analysis (Mann-Whitney U test), when comparing the two active treatment agents against each other, there was no significant difference in blood loss (p = 1.0). However, the blood loss for tranexamic acid was significantly lower than for the control group (p = 0.02). Similarly, losses in the fibrin spray (p=0.02) treatment group were significantly lower than in the controls. Expressed as a percentage difference between median values, losses were 22% lower in the tranexamic group and 32% lower in the fibrin spray group (Figure).

**Figure:**
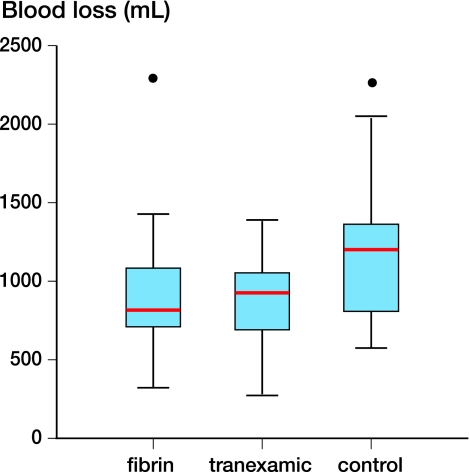
Box plot of blood loss for each of the study groups (in mL), showing median, interquartile range (box), 5 and 95 percentiles (whiskers), and outliers.

## Discussion

Fibrin spray and tranexamic acid have both been reported to reduce blood loss in arthroplasty (Benoni et al. 2001, Ho and Ismail 2003, Husted et al. 2003, Johansson et al. 2005, Niskanen and Korkala 2005, Mawatari et al. 2006, Rajesparan et al. 2009). Prior to the time of commencement of our study, each of the treatments had been used for some patients at the hospital where the study was conducted.

Our method of blood loss calculation was chosen in light of previous work, which has demonstrated that measuring blood loss from swabs and suction drainage can lead to substantial underestimation of the actual loss (Sehat et al. 2004). Thus, we were not surprised to find that our calculated total blood losses were higher than those measured from swabs postoperatively. For this reason, we did not analyze the losses measured from swabs further, but used the more accurate calculated values.

Tranexamic acid reduces blood loss by reducing local fibrinolysis, which is in turn caused by the activation of tissue plasminogen activator (Claeys et al. 2007). Previous studies have found that tranexamic acid reduces blood loss and transfusions following hip arthroplasty (Ho and Ismail 2003, Husted et al. 2003, Garneti and Field 2004, Johansson et al. 2005, Niskanen and Korkala 2005, Rajesparan et al. 2009). It does not lead to an increase in the rates of deep venous thrombosis (Ho and Ismail 2003). It is relatively cheap and has been shown to be cost effective (Johansson et al. 2005).

Fibrin spray is manufactured from human plasma products. It has been shown to be safe and effective in reducing blood loss and requirements for transfusion after hip arthroplasty (Wang et al. 2003, Mawatari et al. 2006). It is considerably costlier than tranexamic acid. The product is processed to remove pathogens and screened to detect contaminants, and has been fully approved and licensed for use in the UK. In common with any blood-derived treatment, there is a theoretical risk of transmission of disease by an unknown vector, a fact that was stated in our patient information sheet. We find it notable that some patients did not wish to participate in the study for this reason. Thus, if fibrin spray were to become widely used in orthopedic surgery, such risks should be included in the informed consent process. Also, in the geographic region in which this study was conducted, if fibrin spray has been used then patients are subsequently excluded from donating blood and bone. Fibrin sealants using autologous donated blood would remove these issues. Such sealants have been shown to be effective in reducing blood loss in hip arthroplasty (Mawatari et al. 2006) but at present they remain a rather specialist treatment. In common with many UK hospitals, we do not have the facilities to prepare and use them.

Interestingly, the Quixil fibrin spray contains tranexamic acid as one of its ingredients. Whilst the manufacturers state that its mechanism of action is due to the presence of human clotting factors, it is possible that the tranexamic acid is responsible for part of its action. To definitively answer this question would require a comparison of topically sprayed (rather than intravenous) tranexamic acid and topical fibrin spray, which was beyond the scope of this study.

One important limitation of our study was the single-blind design. This was necessitated by the fact that we did not have a placebo for the fibrin spray. The manufacture and sterilization of a convincing placebo could not be achieved with the resources available. Also, the ethics committee had expressed concerns that any placebo preparation might expose patients to an unnecessary potential wound contaminant.

Patients in the fibrin group received 10 mL of topical fibrin spray, given in divided doses throughout the operation, which was the manufacturer's recommended dose for this indication. Patients in the tranexamic acid group were given a single 10 mg/kg bolus dose of tranexamic acid intravenously, at induction of anesthesia. The manufacturer did not state a recommended dose for this surgery, so our dose and timing was chosen on the basis of previous studies that have found a 10 mg/kg bolus to be effective in hip arthroplasty when given at induction (Benoni et al. 2001, Garneti and Field 2004). While we confirmed the effectiveness of this dose in our study population, it is worth noting that the same dose has been found to be ineffective if given at the end of surgery (Benoni et al. 2000). Some authors have reported giving a higher dose (Johansson et al. 2005, Claeys et al. 2007) or repeated doses (Ekbäck et al. 2000, Ido et al. 2000, Husted et al. 2003, Niskanen and Korkala 2005), with the aim of prolonging the time at which the drug is maintained at its therapeutic plasma concentration.

When compared to control, both treatments were effective in THR—as we had hypothesized. This is consistent with previous literature regarding the use of either medication individually (Benoni et al. 2001, Ho and Ismail 2003, Husted et al. 2003, Johansson et al. 2005, Niskanen and Korkala 2005, Mawatari et al. 2006, Rajesparan et al. 2009), although at the time of writing we are not aware of any previous direct comparison of both medications in THR.

We conclude that in cemented total hip arthroplasty, blood loss was reduced by the use of either tranexamic acid or fibrin spray when compared to controls. Neither intervention was superior to the other. It is not possible to generalize the results in this study to uncemented hip arthroplasty or to arthroplasty of other joints.

One area in which the two treatments differ greatly is their relative cost. The intravenous preparation of tranexamic acid (Cyklokapron) used in this study costs €1.80 per 5-mL (500-mg) ampoule at the time of writing. The average patient weighing 70 kg, given a single 10 mg/kg dose, would receive 700 mg at a cost of €3.60 (the unused 300 mg being discarded). In contrast, the current price of the fibrin spray (Quixil) preparation is €450. Given the equivalent effects of both medications in this study, together with the cost considerations and theoretical harms from the use of blood-derived products, it would seem prudent to use tranexamic acid in preference to fibrin spray.
